# The decline in tropical land carbon sink drove high atmospheric CO_2_ growth rate in 2023

**DOI:** 10.1093/nsr/nwae365

**Published:** 2024-10-22

**Authors:** Yanchen Gui, Kai Wang, Zhe Jin, Heyuan Wang, Hanzhi Deng, Xiangyi Li, Xiangjun Tian, Tao Wang, Wei Chen, Tengjiao Wang, Shilong Piao

**Affiliations:** Institute of Carbon Neutrality, Sino-French Institute for Earth System Science, College of Urban and Environmental Sciences, Peking University, Beijing 100871, China; Institute of Carbon Neutrality, Sino-French Institute for Earth System Science, College of Urban and Environmental Sciences, Peking University, Beijing 100871, China; Institute of Carbon Neutrality, Sino-French Institute for Earth System Science, College of Urban and Environmental Sciences, Peking University, Beijing 100871, China; State Key Laboratory of Tibetan Plateau Earth System, Resources and Environment (TPESRE), Institute of Tibetan Plateau Research, Chinese Academy of Sciences, Beijing 100101, China; School of Computer Science, Peking University, Beijing 100871, China; Institute of Computational Social Science, Peking University (Qingdao), Qingdao 266555, China; School of Computer Science, Peking University, Beijing 100871, China; Institute of Computational Social Science, Peking University (Qingdao), Qingdao 266555, China; Institute of Carbon Neutrality, Sino-French Institute for Earth System Science, College of Urban and Environmental Sciences, Peking University, Beijing 100871, China; State Key Laboratory of Tibetan Plateau Earth System, Resources and Environment (TPESRE), Institute of Tibetan Plateau Research, Chinese Academy of Sciences, Beijing 100101, China; University of Chinese Academy of Sciences, Beijing 101408, China; State Key Laboratory of Tibetan Plateau Earth System, Resources and Environment (TPESRE), Institute of Tibetan Plateau Research, Chinese Academy of Sciences, Beijing 100101, China; School of Computer Science, Peking University, Beijing 100871, China; Institute of Computational Social Science, Peking University (Qingdao), Qingdao 266555, China; School of Computer Science, Peking University, Beijing 100871, China; Institute of Computational Social Science, Peking University (Qingdao), Qingdao 266555, China; Institute of Carbon Neutrality, Sino-French Institute for Earth System Science, College of Urban and Environmental Sciences, Peking University, Beijing 100871, China; State Key Laboratory of Tibetan Plateau Earth System, Resources and Environment (TPESRE), Institute of Tibetan Plateau Research, Chinese Academy of Sciences, Beijing 100101, China

**Keywords:** carbon budget, land carbon sink, atmospheric CO_2_ growth rate, artificial intelligence, El Niño

## Abstract

Atmospheric CO_2_ growth rate (CGR), reflecting the carbon balance between anthropogenic emissions and net uptake from land and ocean, largely determines the magnitude and speed of global warming. The CGR at Mauna Loa Baseline Observatory reached a record high in 2023. We quantified major components of the global carbon balance for 2023, by developing a framework that integrated fossil fuel CO_2_ emissions data and an atmospheric inversion from the Global ObservatioN-based system for monitoring Greenhouse GAses (GONGGA) with two artificial intelligence (AI) models derived from dynamic global vegetation models. We attributed the record high CGR increase in 2023 compared to 2022 primarily to the large decline in land carbon sink (1803 ± 197 TgC year^−1^), with minor contributions from a small reduction in ocean carbon sink (184 TgC year^−1^) and a slight increase in fossil fuel emissions (24 TgC year^−1^). At least 78% of the global decline in land carbon sink was contributed by the decline in tropical sink, with GONGGA inversion (1354 TgC year^−1^) and AI simulations (1578 ± 666 TgC year^−1^) showing similar declines in the tropics. We further linked this tropical decline to the detrimental impact of El Niño-induced anomalous warming and drying on vegetation productivity in water-limited Sahel and southern Africa. Our successful attribution of CGR increase within a framework combining atmospheric inversion and AI simulations enabled near-real-time tracking of the global carbon budget, which had a one-year reporting lag.

## Teaser

This study provides a near-real-time tracking of the global carbon budget using atmospheric CO_2_ inversion and artificial intelligence models based on ecosystem process models. The record high CO_2_ growth rate in 2023 is attributed to the large decline in tropical land carbon sink.

## INTRODUCTION

**Figure 1.**
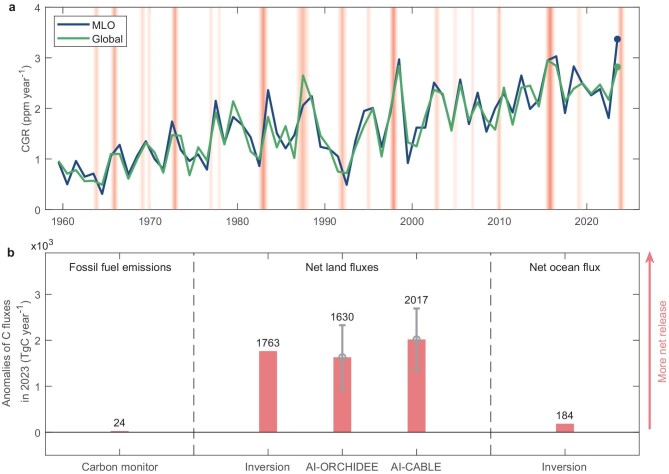
Contributions of net land flux, net ocean flux and fossil fuel emissions to the high CO_2_ growth rate (CGR) in 2023. (a) CGR variations from 1959 to 2023 in the MLO station and global mean estimate based on the MBL observing network (see Methods). The red shaded areas represent El Niño events, with the darker color indicating a stronger El Niño. (b) Anomalies of each carbon flux in 2023 compared to 2022. Positive values mean more net release into the atmosphere. Fossil fuel emissions are estimated using the Carbon Monitor data set. We ran an atmospheric inversion to estimate the inversion-based net land flux and net ocean flux. And we also used the AI-ORCHIDEE and AI-CABLE models to estimate two sets of net land fluxes (see Methods), each consisting of five ensemble members. The error bars for each AI model indicate the standard deviation across these ensemble members.

The growth rate of atmospheric CO_2_ (CGR) reflects the balance between anthropogenic carbon emissions and net carbon uptake from land and ocean, whose interannual variability is tightly related to climate anomalies induced by the El Niño-Southern Oscillation (ENSO) [1,2]. In 2023, the CGR reached 3.37 ppm year^−1^ at Mauna Loa Baseline Observatory (MLO), the highest value since records began in 1959 (Fig. 1a). With this exceptionally high increase in CGR, the annual mean atmospheric CO_2_ concentration at MLO exceeded 420 ppm for the first time, reaching 1.5 times the level at the start of the industrial era [3]. It is crucial then to attribute this record-high CGR increase to different components of the global carbon balance. Since 2006, the Global Carbon Project has produced an annual global carbon budget that reports changes in global carbon emissions and sinks [2]. But there is a one-year delay in the budget estimation, leading to the global carbon budget for 2023 still being unknown in the second half of 2024. The detailed and near-real-time tracking of global carbon budget would therefore be highly valuable.

To address this gap, we firstly integrated an atmospheric inversion from the Global ObservatioN-based system for monitoring Greenhouse GAses (GONGGA) with two artificial intelligence (AI) models based on dynamic global vegetation models (DGVMs) to estimate land and ocean carbon sinks in 2023. Through further combination with fossil fuel CO_2_ emissions, we analyzed changes in the global carbon balance in 2023 relative to 2022 and investigated causes of this record high CGR increase in 2023.

## RESULTS

### A reduced land carbon sink drove record high CGR increase in 2023

The CGR at MLO in 2023 increased by 1.56 ppm year^−1^ when compared with 2022 (Fig. 1a). Based on CO_2_ observations at the well-mixed marine boundary layer (MBL) air representative stations [4], the global mean CGR in 2023 reached 2.82 ppm year^−1^, an increase of 0.65 ppm year^−1^ relative to 2022, and came very close to the levels observed during the two strongest El Niño events (1997–1998, 2.84 ppm year^−1^; and 2015–2016, 2.95 ppm year^−1^) in the past three decades [5] (Fig. 1a).

To investigate the factors contributing to this exceptionally high CGR increase in 2023, we assessed anomalies of land carbon flux, ocean carbon flux and fossil fuel carbon emissions, with positive and negative values indicating net carbon release and uptake, respectively. With GONGGA atmospheric inversion, the global net land sink substantially declined by 1763 TgC year^−1^ in 2023 (1196 TgC year^−1^) when compared with 2022 (2959 TgC year^−1^) (Fig. 1b). Such inversion-based decline in the global net land sink is consistent with that from two AI models (1630 ± 698 TgC year^−1^ and 2017 ± 673 TgC year^−1^) (Fig. 1b). Here we used an AI system to train AI models (AI-ORCHIDEE and AI-CABLE) using outputs of two DGVMs (ORganizing Carbon and Hydrology in Dynamic EcosystEms, ORCHIDEE; Community Atmosphere-Biosphere Land Exchange, CABLE), and then forced the AI models, with time-varying climate and CO_2_ concentration, to predict the land carbon sinks for each year (see Methods). We demonstrated that the AI system can swiftly capture the spatial-temporal patterns of land carbon sink. It harmonizes the scale differences across 6-hourly climate variables, annual atmospheric CO_2_ concentrations and monthly carbon fluxes, and learns interregional heterogeneity in long-term trends and short-term dynamics (see Methods). The AI-based approach did not include land-use carbon emissions such as deforestation-induced fire emissions, which were expected to change little between the two adjacent years [6]. Notably, the decline of the global land sink in 2023 relative to 2022 is comparable to, if not greater than, the detrended anomalies of global land sink during the 1997–1998 and 2015–2016 El Niño events [7,8]. By contrast, fossil fuel carbon emissions have a slight increase (24 TgC year^−1^) and ocean carbon sink has decreased by 184 TgC year^−1^ in 2023 when compared with 2022 (Fig. 1b), suggesting that the observed exceptionally high CGR increase in 2023 is due to a massive decline in global land carbon sink.

### Regional contributions to the decline in global land carbon sink in 2023

**Figure 2.**
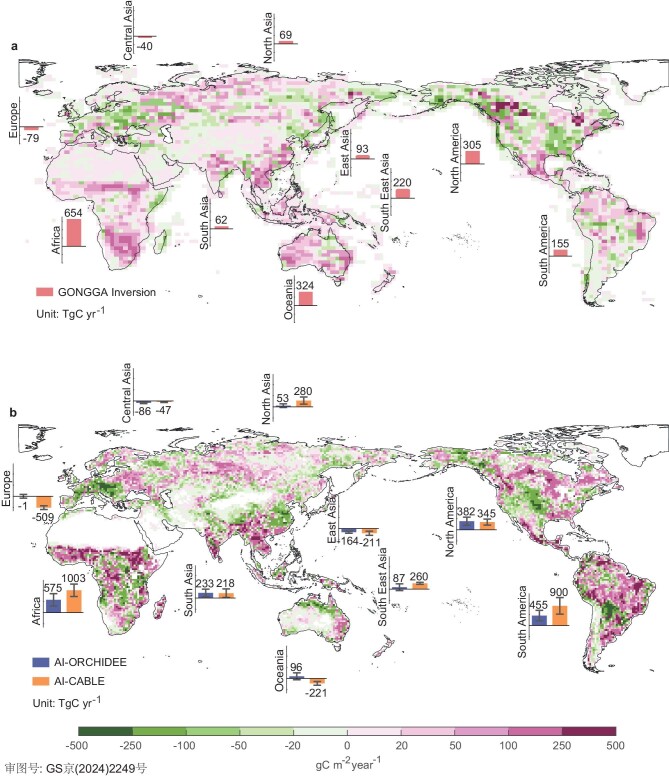
Spatial patterns and regional contributions of net land flux anomalies in 2023. (a) Spatial pattern of inversion-based land sink anomalies in 2023 compared to 2022. The column of the histogram shows the anomalies of inversion-based net land sink for each RECCAP region. Positive values suggested more carbon release into the atmosphere. (b) Same as for (a), but replacing the land carbon sink with the average of AI-ORCHIDEE and AI-CABLE estimates. The two columns of the histogram show the anomalies of land sink estimated by AI-ORCHIDEE and AI-CABLE for each region, respectively, with uncertainties indicating the standard deviation of their five ensemble members.

Next, we quantified regional contributions to the decline in global land carbon sink in 2023 relative to 2022, by classifying the global land into 10 regions according to the REgional Carbon Cycle Assessment and Processes (RECCAP) project [9]. Based on GONGGA atmospheric inversion, most of the tropics witnessed a widespread decline in net carbon uptake in 2023 relative to 2022 (Fig. 2a), which is due to enhanced net ecosystem exchange (NEE) rather than carbon emissions due to fire disturbances (Fig. S1). The tropical land carbon sink (Africa, South America, Oceania and South East Asia) declined by 1354 TgC year^−1^ relative to 2022, accounting for 78% of the global decline in land carbon sink. This inversion-based decline in tropical land carbon sink is also comparable to those derived from AI-ORCHIDEE (1213 ± 546 TgC year^−1^) and AI-CABLE models (1942 ± 607 TgC year^−1^). Despite uncertainties in regional estimates and the spatial pattern of carbon sink anomalies, both atmospheric inversion and AI models based on DGVMs agreed that tropical land mainly drove the decline of the global land carbon sink in 2023 relative to 2022 (Fig. 2a and Fig. S2).

By contrast, according to GONGGA atmospheric inversion, the northern extra-tropical land carbon sink only declined by 410 TgC year^−1^ in 2023 relative to 2022, which contributed much less than tropical land to the global decline in land carbon sink. In 2023, there were increased fire-induced carbon emissions that were primarily located in Canada [10] (Fig. S1b). For example, the record wildfires caused 676 TgC year^−1^ more carbon emissions over boreal North America in 2023 than 2022 [11]. But this fire-induced decline in carbon sink was largely offset by enhanced carbon sink in other regions such as the USA (Fig. S1a), thereby leading to a muted decline in northern carbon sink. Note that the fire-induced carbon loss was not considered in AI models due to the absence of fire processes in DGVMs. However, the inversion-based decline remains comparable to AI-ORCHIDEE, which simulated a decrease of 417 ± 377 TgC year^−1^ in 2023 compared to 2022. This similarity arises because anomalous warmer conditions in 2023 increased ecosystem respiration in AI model simulations, resulting in a decrease in carbon sink of similar magnitude to that from fires.

The greater decline of land carbon sink in the tropics than in northern extra-tropics would explain why CGR at MLO increased more than its global mean based on MBL stations in 2023 relative to 2022 (Fig. 1a). MLO is located close to the equator, and the interannual variability of CGR at MLO was shown to be strongly correlated with variability of tropical land carbon sink [12–14]. Therefore, the record high CGR increase at MLO could be attributed to the substantial decline in tropical land carbon sinks (Fig. 2). In contrast, the global mean CGR from MBL stations would be much more influenced by land CO_2_ fluxes in the northern extra-tropics, and a small reduction in northern extra-tropical land carbon sink thereby contributes to a relatively lower CGR increase at the global mean level than at MLO (Fig. 1a).

### El Niño-induced decline in tropical land sink in 2023

Both GONGGA atmospheric inversion and AI models based on DGVMs agreed that Africa contributed more to the decline in tropical land sink than the other three tropical regions (South America, Oceania and South East Asia) in 2023 relative to 2022. For example, GONGGA, AI-ORCHIDEE and AI-CABLE suggest that Africa contributed 48%, 48 ± 21% and 54 ± 14% of the decline in land carbon sink in four tropical regions, respectively (Fig. 2). Both inversion and AI models simulated that the African carbon sink decline mainly occurred in the Sahel and southern Africa (Fig. 2a and Fig. S2), although the decline in southern Africa was relatively small in AI models (Fig. S2). We further analyzed AI-model-derived anomalies of the two main carbon cycle components, and found that the decline in carbon sink in 2023 was attributed more to decreased gross primary production (GPP) than enhanced terrestrial ecosystem respiration (TER) (Figs S3 and S4). Such AI-model-based GPP decline across the Sahel and southern Africa (Fig. S3) was also recorded by solar-induced chlorophyll fluorescence (SIF) observations in near real time (Fig. S5).

**Figure 3.**
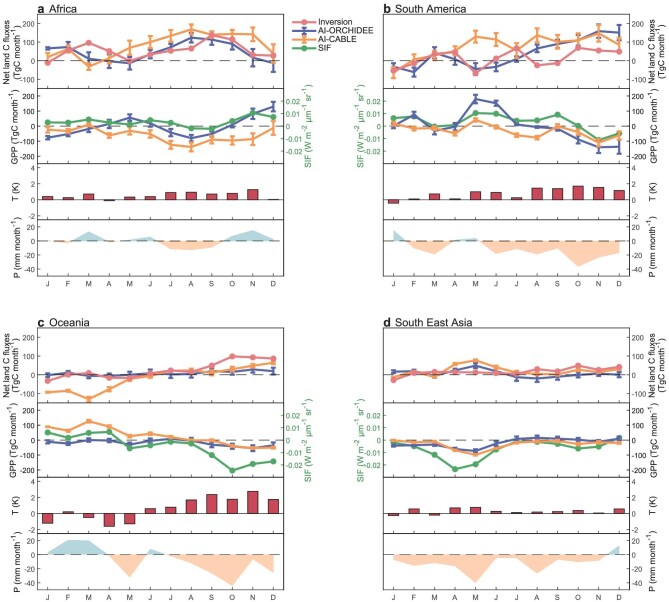
Monthly anomalies of net land flux, vegetation productivity, temperature and precipitation in (a) Africa, (b) South America, (c) Oceania and (d) South East Asia in 2023. In each panel, the first subplot shows the anomalies of land carbon sink, estimated by inversion, AI-ORCHIDEE and AI-CABLE, with uncertainties of the latter two representing the standard deviation of their five ensemble members. Positive values of net land carbon fluxes mean more carbon release into the atmosphere. The second subplot shows GPP anomalies estimated by AI-ORCHIDEE and AI-CABLE with uncertainties, and area weighted average SIF anomalies. The third and fourth subplots show area weighted average temperature and precipitation anomalies, respectively.

The notable GPP decline across the water-limited regions of the Sahel and southern Africa [15] in 2023 could be mainly attributed to a widespread decline in the total water storage relative to 2022 (Fig. S6) [16]. This continental-scale picture arises from El Niño-induced drying (Figs S7 and S8), as well as anomalous warming in the second half of 2023 (Fig. 3a and Fig. S9), which further exacerbated water deficits by enhancing the evaporation. Except for Africa, El Niño-associated drying and warming-exacerbated water deficits in the second half of 2023 also led to declines in GPP and carbon sinks in South America, Oceania and South East Asia (Fig. 3b–d). It is interesting to note that South East Asia experienced an even larger decline in GPP and carbon sinks from April to June of 2023 than during the second half of 2023 (Fig. 3d and Fig. S10), suggesting the effects of climate modes other than El Niño on the carbon sink in South East Asia [17–19].

This notable decline in tropical carbon sinks in 2023 is even greater than that in 2015–2016, and was accompanied by a much stronger El Niño event [7] (Fig. S11), tentatively suggesting an enhanced apparent sensitivity of tropical land carbon sink to El Niño events under global warming [20]. Although the decline in tropical land carbon sinks primarily occurred in Africa during both El Niño events [21], it predominantly affected semi-arid ecosystems in 2023 while mainly impacting tropical forests in 2015–2016 [21]. Such spatially variable ecosystem responses were due to the diversity of El Niño events and their teleconnection patterns [22,23].

## CONCLUSIONS AND PERSPECTIVES

Here we integrated atmospheric CO_2_ inversion with AI models to estimate the global terrestrial carbon balance for the year 2023. We showed that the large decline in tropical land carbon sinks is primarily responsible for the reduction in the global land carbon sink, thereby resulting in the record high CGR observed in 2023 at MLO. The warmer and drier conditions associated with El Niño events have reduced tropical land sinks in water-limited regions especially, contributing to a substantial decline in tropical land sinks. The decline in 2023 is 745 TgC year^−1^ greater than the decline that was due to the 2015–2016 El Niño event. Since the El Niño did not subside by the end of 2023, the resultant decline in tropical carbon sinks is likely to exceed what was observed in 2023. We therefore provided a robust framework for near-real-time estimation of terrestrial land carbon sinks. Additionally, the onset of an El Niño event in 2023 may enhance ocean carbon uptake in the tropical Pacific. This increase was seasonally compensated by the preceding La Niña, resulting in a minor change in annual ocean carbon sink. Although the inverted ocean carbon sink from GONGGA was found to be comparable to those from other state-of-the-art inversion systems [24], we should assimilate both atmospheric CO_2_ retrievals and *in situ* observations in the GONGGA inversion system to obtain a more accurate estimate of ocean carbon sinks.

The atmospheric inversion could provide robust estimates of land carbon sinks at global and hemispherical scales [6,25]. The close correspondence of AI-model-based estimates with inversion results demonstrates the capacity of AI models to convincingly capture the detrimental impact of hot and dry conditions on land carbon sinks during 2023 at the global and continental scales. After evaluation against satellite observations, AI models were also shown to well capture carbon cycling processes at regional scales, where the uncertainties of the inversion estimates would grow rapidly [26–28]. For example, in contrast to the atmospheric inversion, which reported little change in land carbon sinks over the mid-western USA during 2023 (Fig. 2a), the AI models suggested enhanced carbon sinks due to enhanced vegetation productivity. This increased productivity was consistent with SIF and vegetation greenness observations [29] (Fig. S5). AI models have inevitable limitations that are inherent in dynamic global vegetation models, including the lack of fire-induced carbon emissions, such as the deforestation-induced fire emissions during El Niño; deficiencies in the simulation of forest mortality; and inaccuracies in modeling carbon cycle responses to extreme climates [30]. These limitations highlight the significant areas for enhancement in future model development. We call for more observations to refine the AI model, which will significantly enhance our capacity to assess the global carbon cycle in a timely and more precise manner in the future.

## METHODS

### CO_2_ growth rate

The annual mean CGR data for MLO since 1959 are available at gml.noaa.gov/ccgg/trends/data.html (last accessed: 11 June 2024), provided by National Oceanic & Atmospheric Administration (NOAA)/Global Monitoring Laboratory (GML) and Scripps Institution of Oceanography [31]. For observations at MLO, the most recent November–February average is used as the estimate for 1 January of the current year. The annual mean CGR for the current year is calculated by subtracting the estimates for 1 January of the current year and the following year. Note that due to the eruption of the Mauna Loa Volcano, measurements from MLO were temporarily suspended, and observations from December 2022 to July 2023 are from a station at the Maunakea Observatories, which is ∼21 miles north of the MLO station and has an elevation of 4199 masl.

The annual mean CGR data based on globally averaged MBL surface data are available at gml.noaa.gov/ccgg/trends/gl_data.html (last accessed: 11 June 2024) [32,33]. The calculation of MBL global annual mean CGR is essentially the same as that for MLO observations, but the December to January average is used as the estimate for 1 January. We noted that using the December to January average or November to February average did not change the anomaly of CGR in 2023 relative to 2022. Since data from the MBL observing network began in 1980, the pre-1980 values of global CGR are complemented by the annual difference in mean concentration at MLO and the South Pole station.

### Solar-induced chlorophyll fluorescence data

SIF has shown great potential to monitor the photosynthetic activity of terrestrial ecosystems. The GOSIF data product was developed based on discrete OCO-2 SIF soundings, moderate resolution imaging spectroradiometer (MODIS) and meteorological reanalysis data, and upscales to a global product at 0.05° resolution [34]. Here we used the monthly SIF data product as an auxiliary reference for GPP.

### Terrestrial water storage data

The TWS data were obtained from the Gravity Recovery and Climate Experiment (GRACE) mission, launched in March 2002 under the National Aeronautics and Space Administration (NASA) Earth System Science Pathfinder Program. Here we investigated TWS variations in 2023 relative to 2022 using the Center for Space Research (CSR) GRACE and Gravity Recovery and Climate Experiment Follow-On (GRACE-FO) data product [16] based on newly RL06.3 mascon solutions.

### Climate data

We used global gridded air temperature and total precipitation data sets from the ERA5-Land reanalysis data products [35], which have a resolution of 0.1° × 0.1°. For the air temperature, we downloaded the monthly average reanalysis 2-m temperature data. Due to the lack of monthly average reanalysis total precipitation in 2023 (last accessed: 12 June 2024), we used hourly total precipitation data and converted them to a monthly average. When training our AI models, we used CRUJRAv2.4 meteorological reanalysis [36] to be consistent with TRENDY-v12 models [6]. The climate variables from CRUJRAv2.4 meteorological reanalysis we used include temperature at 2 m, maximum temperature at 2 m, minimum temperature at 2 m, total precipitation, specific humidity, downward solar radiation flux, downward long wave radiation flux, pressure, zonal component of wind speed, and meridional component of wind speed.

### ENSO indices

The ENSO is closely correlated with the interannual variations of CGR. Here we used the indices of the three-month running mean of sea surface temperature (ERSST.v5 data) anomalies in the Niño 3.4 region (5°N–5°S, 120°W–170°W), which is called the Oceanic Niño Index (ONI). El Niño (La Niña) is defined as an ONI value above (below) the threshold of +0.5°C (−0.5°C) for five or more consecutive months.

### Atmospheric CO_2_ inversion

We generated a set of monthly net land biosphere productivity (NBP) and ocean exchange fluxes (2° latitude × 2.5° longitude) from 2022 to 2023, utilizing the GONGGA atmospheric inversion system [24,25], which has been used in the most recent estimates of the global carbon budget [6,37]. The inversion that was run for this study assimilated the column-averaged dry air mole fraction of CO_2_ data of OCO-2 retrievals (V11.1) [38], and it used the Global gRidded dAily CO_2_ Emissions Dataset (GRACED) for fossil fuel emissions [39] and Global Fire Emissions Database (GFED 4.1s) for fire emissions [40]. Note that the output of the GONGGA inversion are the NEE fluxes. The inversion-based net land sink used in this study is the sum of the inversion output NEE and GFED fire emissions.

### Artificial intelligence system to simulate land carbon fluxes

We have trained an AI system for spatiotemporal variation learning, which discerns the underlying projection from climate variables and CO_2_ concentration to GPP and NBP outputs of TRENDY-v12 models in S2 simulation across the global land for the past 120 years. Specifically, two AI-based predictive models have been generated by the system, called AI-ORCHIDEE and AI-CABLE, respectively leveraging the outputs of ORCHIDEE and CABLE-POP models in TRENDY-v12. To be consistent with TRENDY-v12 models [6], climate variables were obtained from CRUJRAv2.4 meteorological reanalysis [36]. The AI system can effectively address the challenge of reconciling the varied timescales inherent in carbon fluxes (monthly), climate variables (6 h) and CO_2_ concentration (yearly). Moreover, it simultaneously captures the long-term trends, short-term dynamics and synergistic effects of multi-variables on carbon fluxes, taking into account the interregional heterogeneity (Piao *et al.*, in prep.).

To optimize the parameters of the AI model, we perform 5-fold cross-year validation. Specifically, the past 120 years (1903–2022) were randomly shuffled and divided into 5 groups, each with a length of 24 years. For each running fold, four groups were used for training and the remaining one was used for testing. The training and test processes were done five times to ensure that each group was used for a test, thus resulting in five ensemble members of each AI model. For each ensemble member, carbon fluxes for 24 years within 1903–2022 were predicted. Consequently, by pooling the prediction results of 5 ensemble members altogether, the AI model can reproduce complete GPP and NBP variations in the past 120 years (Fig. S12).

Our goal is to use the well-trained AI models to simulate (predict) the GPP and NBP for 2023 by incorporating climate and CO_2_ data in that year. We validated the AI model's predictive accuracy, treating GPP and NBP in 2022 as the target. Specifically, we trained the AI models using carbon fluxes, climate variables and CO_2_ concentration during the period of 1903–2021, and then used these models to predict the monthly GPP and NBP in 2022. We found that the AI models could well capture not only the annual GPP and NBP at the global scale (Table S1) but also the seasonal variations of GPP and NBP at grid scale in 2022 (Figs S13–S16). In addition, we found that the AI-CABLE and AI-ORCHIDEE point to a significant anomaly, estimated at 0.75 PgC year^−1^ and 0.58 PgC year^−1^, in the tropical land carbon sink during the 2015–2016 El Niño (Fig. S11). These values are within the range of estimation provided by Bastos *et al.* [7]. This indicates that the AI models are well-equipped to simulate (predict) the variations in GPP and NBP for the year 2023 if climate and CO_2_ data in 2023 are available.

To simulate (predict) the variations in GPP and NBP for the year 2023, we first calculated the yearly CO_2_ concentration in 2023 following the TRENDY protocol (https://blogs.exeter.ac.uk/trendy/protocol/). Specifically, the monthly CO_2_ concentration in 2023 was obtained by calculating the average from the MLO and South Pole Observatory (SPO), provided by NOAA's Earth System Research Laboratory. Then the yearly CO_2_ concentration was calculated as the average of the monthly values. For climate variables, given that the Climatic Research Unit and Japanese reanalysis (CRUJRA) meteorological reanalysis is only annually updated and is aligned with the Climatic Research Unit gridded Time Series (CRU TS) data set, we used temperature and precipitation provided by CRU TSv4.08 for 2023 [41]. We then calculated the 6-h maximum temperature and minimum temperature in 2023 based on the difference of the monthly average of temperature with the monthly average of maximum temperature and monthly average of minimum temperature. We also used cloud cover provided by CRU TS to calculate the downward solar radiation flux in 2023 because the downward solar radiation flux of CRUJRA is aligned with cloud cover of CRU TS. To do that, a linear fit between downward solar radiation flux and cloud cover was conducted for each month. Then according to the linear fit and cloud cover in 2023 and 2022, the ratio of downward solar radiation flux in 2023 and 2022 for each month was obtained. After that, the downward solar radiation flux in 2023 was obtained with that in 2022. For downward long-wave radiation flux which is not included by the CRU TS data set, we used hourly ERA5 reanalysis to calculate the 6-h values. Given the bias of downward long-wave radiation flux between ERA5 and CRUJRA, we calculated the multi-year averages of monthly downward long-wave radiation flux from ERA5 and CRUJRA for the period of 1991–2020 and then calculated the ratios of the multi-year averages between two data sets for each month. With these ratios, we could adjust the downward long-wave radiation flux in 2023 from ERA5 to be more consistent with that from CRUJRA. For pressure and wind speed, we used the ERA5-Land reanalysis. Specific humidity was calculated using pressure and vapor pressure, which was estimated by dew point temperature from ERA5-Land reanalysis. Therefore, with the CO_2_ concentration and climate variables in 2023, we could simulate the GPP and NBP in this year using the AI-ORCHIDEE and AI-CABLE models. The uncertainty of output of the AI-ORCHIDEE model or AI-CABLE model indicates the standard deviation of results of five ensemble members.

## Supplementary Material

nwae365_Supplemental_File

## Data Availability

The OCO-2 retrievals are available at disc.gsfc.nasa.gov/datasets?page=1&keywords=OCO-2. The carbon monitor fossil fuel emissions data set is available at https://carbonmonitor.org/ and the GRACED fossil fuel emissions data set is available at carbonmonitor-graced.com. The GFED 4.1s fire emissions data set is available at geo.vu.nl/∼gwerf/GFED/GFED4/. The GOSIF data product is available at globalecology.unh.edu/data/GOSIF.html. The ERA5-Land monthly averaged data is available at cds.climate.copernicus.eu/cdsapp#!/dataset/10.24381/cds.68d2bb30?tab=overview, and the hourly data are available at cds.climate.copernicus.eu/cdsapp#!/dataset/reanalysis-era5-land?tab=overview. The ONI indices for ENSO identification are available at https://www.ncei.noaa.gov/access/monitoring/enso/sst. The GRACE/FO TWS data used in this study are available at https://www2.csr.utexas.edu/grace/RL06_mascons.html.
