# Supplementary material for: The decline in tropical land carbon sink drove high atmospheric CO2 growth rate in 2023
Source: Natl Sci Rev. 2024 Oct 22;11(12):nwae365. doi: 10.1093/nsr/nwae365 (PMC11646126; doi:10.1093/nsr/nwae365)
Supplement: nwae365_Supplemental_File [file nwae365_supplemental_file.docx]

Supplementary Information for

**The decline in tropical land carbon sink drove high atmospheric CO_2_ growth rate in 2023**

Gui et al.

This file includes:

Supplementary Table 1, and Supplementary Figures 1-16

**Supplementary Tables**

**Supplementary Table 1.** **The global NBP and GPP estimated by ORCHIDEE and CABLE of TRENDY-v12, and by AI-ORCHIDEE and AI-CABLE (Unit: PgC year^-1^) in 2022.** The uncertainties of the AI models mean the standard deviation of the 5 ensemble members. In the AI estimate, we trained the AI models using carbon fluxes, climate variables, and CO_2_ concentration during the period of 1903−2021, and then used these models to predict the GPP and NBP in 2022.

| **Variable** | **AI models** | **TRENDY-v12** |
| --- | --- | --- |
| ORCHIDEE NBP | 2.62 ± 0.55 | 2.74 |
| ORCHIDEE GPP | 118.51 ±0.73 | 118.68 |
| CABLE NBP | 3.22 ± 0.25 | 3.12 |
| CABLE GPP | 126.58 ± 0.13 | 126.40 |

**Supplementary Figures**


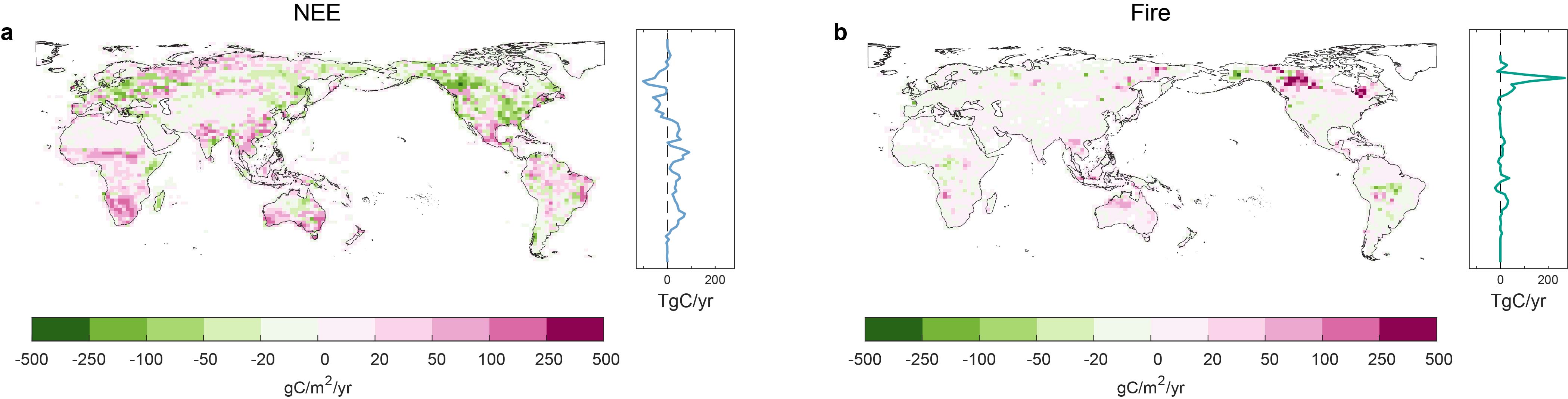


**Supplementary Figure 1 Spatial patterns of net ecosystem exchange (NEE) and fire emissions anomalies in 2023.** Through the atmospheric inversion (see Methods), we obtained the annual estimate of (**a**) NEE and (**b**) fire emissions anomalies in 2023 relative to 2022, with positive values mean more carbon release into the atmosphere. The right panel shows the aggregates of NEE (fire emissions) anomalies every 2° in the latitudinal direction.


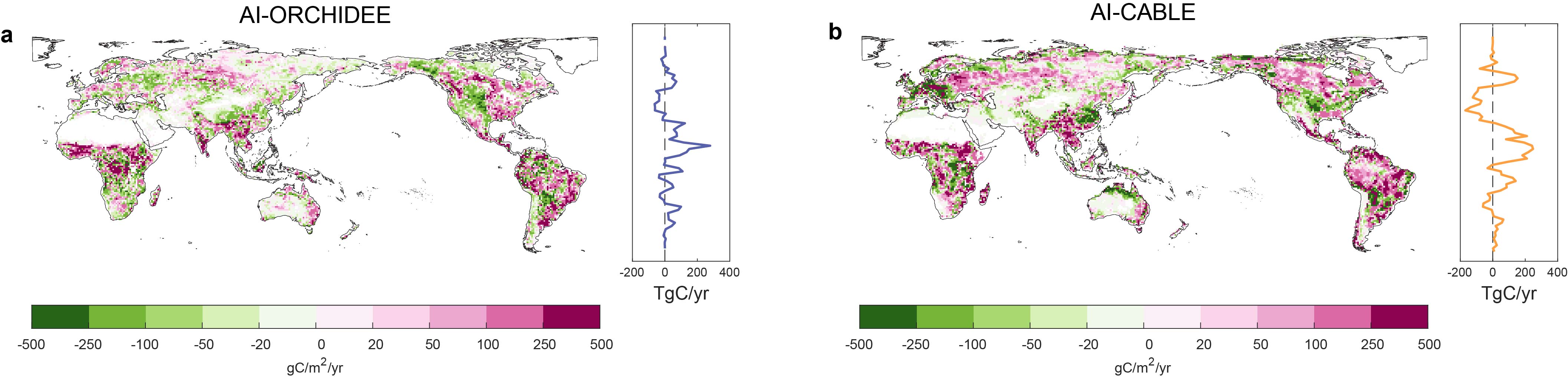


**Supplementary Figure 2 Spatial patterns of land carbon flux anomalies in 2023.** Through the AI-DGVM models (see Methods), we obtained the annual anomalies of net land carbon sink in 2023 relative to 2022 estimated by (**a**) AI-ORCHIDEE and (**b**) AI-CABLE, with positive values mean more carbon release into the atmosphere. The right panel shows the aggregates of land carbon sink anomalies every 2° in the latitudinal direction.


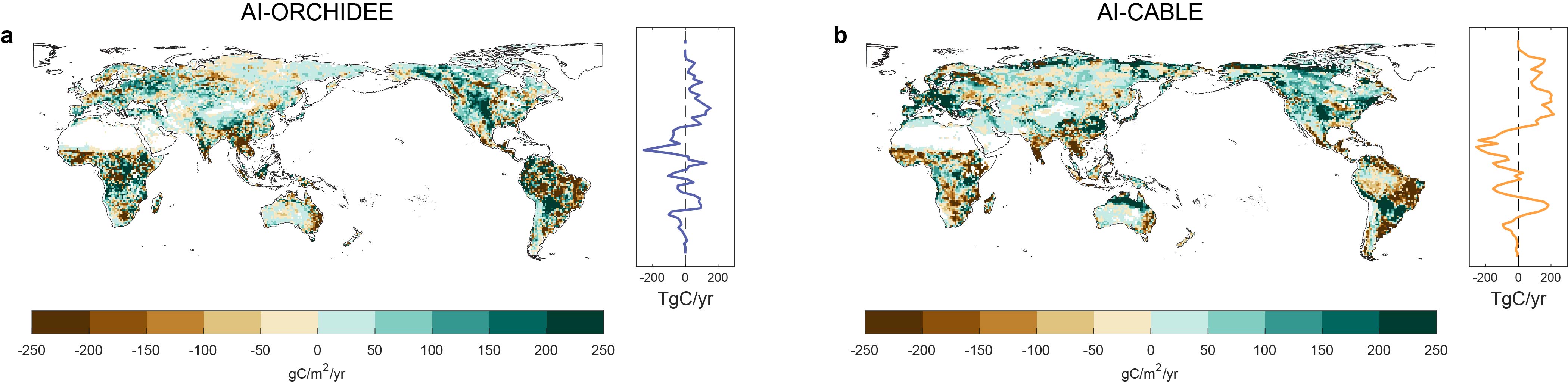


**Supplementary Figure 3 Spatial patterns of gross primary productivity (GPP) anomalies in 2023.** Spatial patterns of GPP in 2023 relative to 2022 estimated by (**a**) AI-ORCHIDEE and (**b**) AI-CABLE, with positive values mean more carbon uptake by the terrestrial ecosystem. The right panel shows the aggregates of GPP anomalies every 2° in the latitudinal direction.


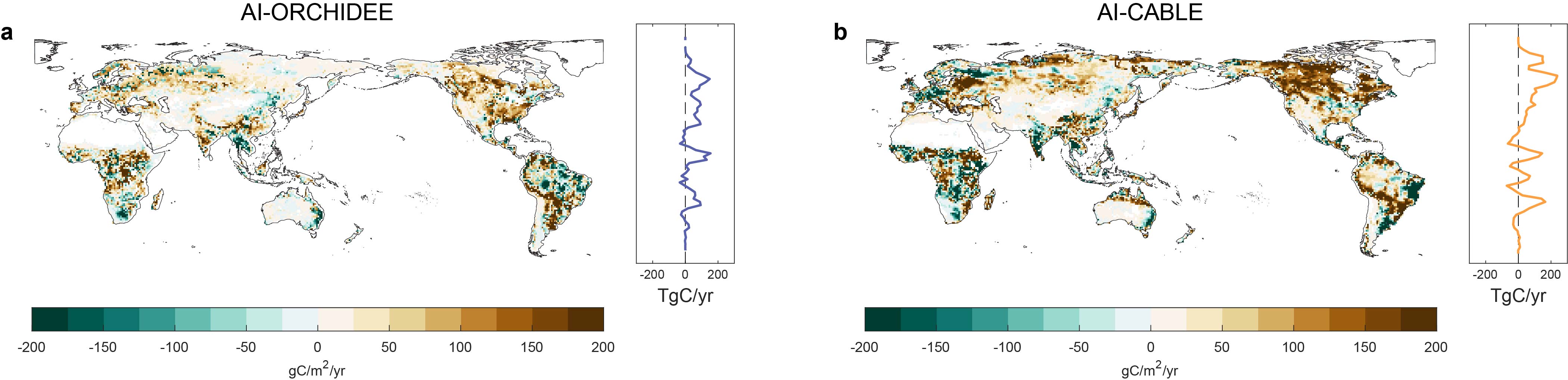


**Supplementary Figure 4 Spatial patterns of total ecosystem respiration (TER) anomalies in 2023.** Spatial patterns of TER in 2023 relative to 2022 estimated by (**a**) AI-ORCHIDEE and (**b**) AI-CABLE, with positive values mean more carbon release into the atmosphere. The right panel shows the aggregates of TER anomalies every 2° in the latitudinal direction.


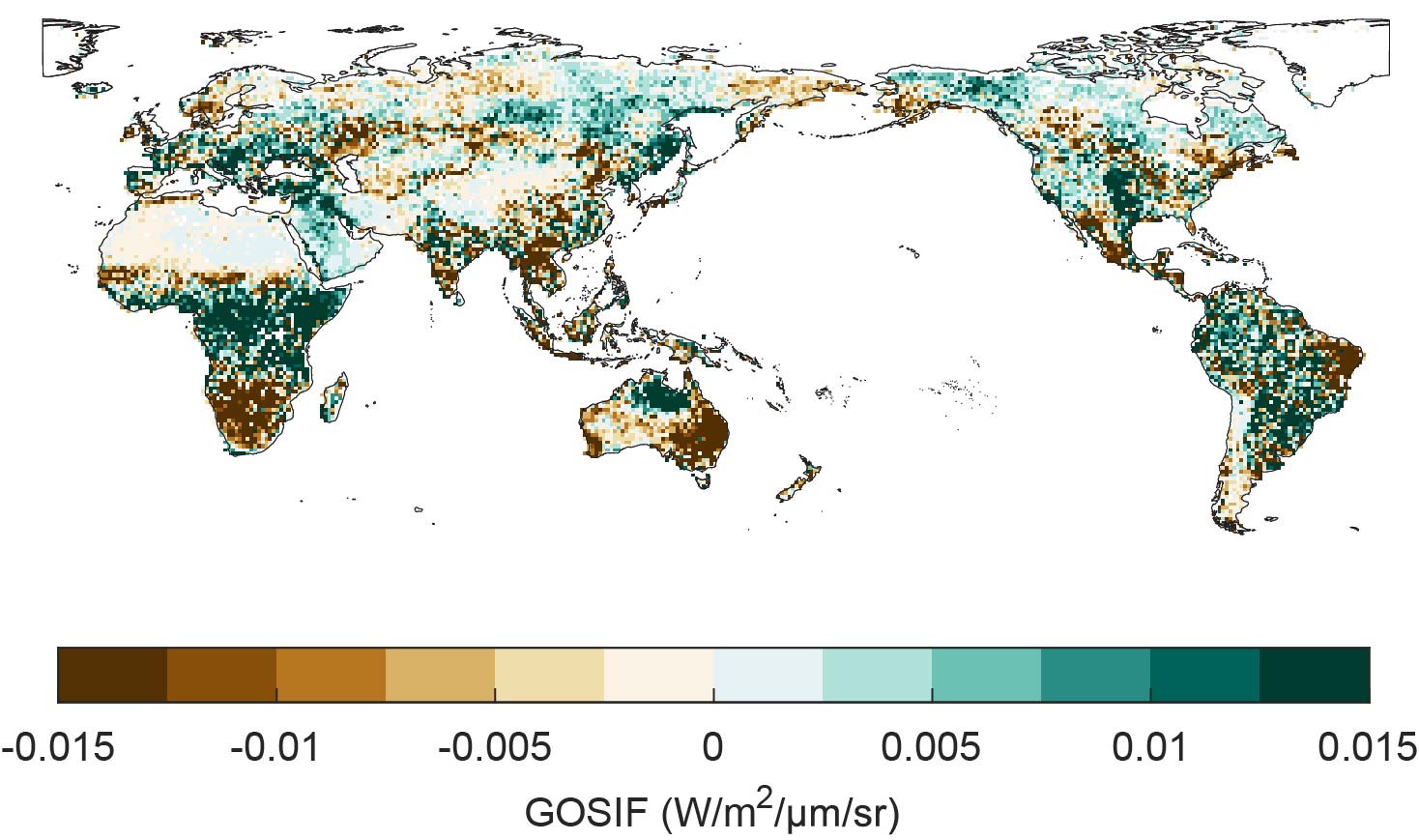


**Supplementary figure 5 Spatial pattern of annual mean GOSIF in 2023 relative to 2022.**


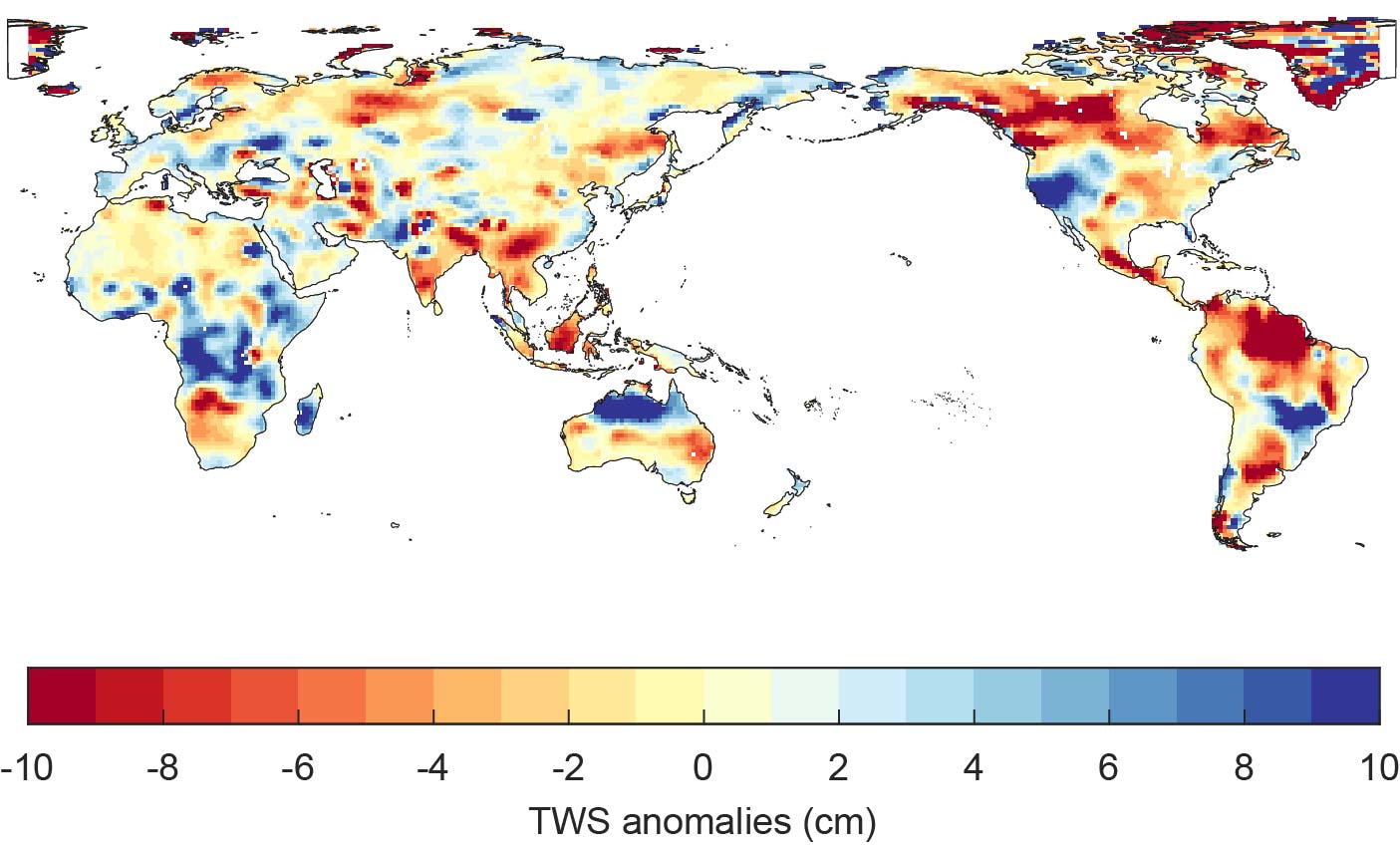


**Supplementary figure 6 Spatial patterns of annual mean TWS in 2023 relative to 2022.**


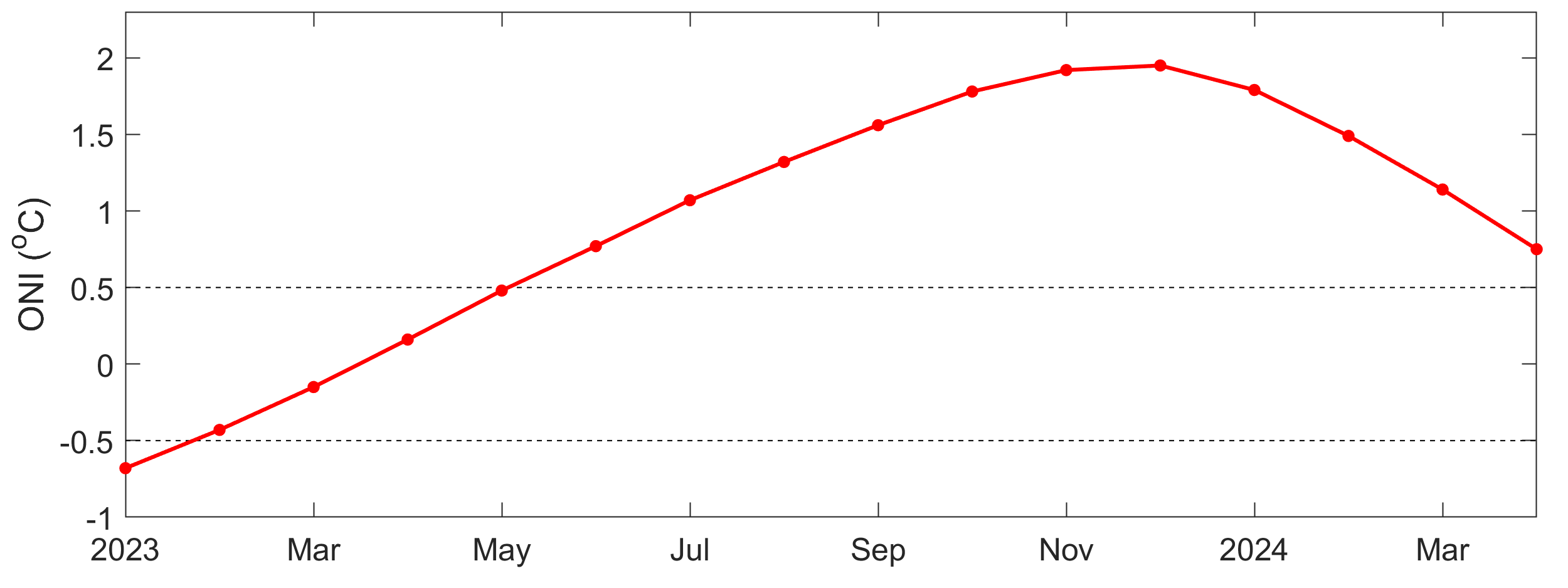


**Supplementary Figure 7 Oceanic Nino Index (ONI) from January 2023 to April 2024.** We utilized ONI indices to define El Niño in 2023, and El Niño is identified as ONI above the threshold of 0.5°C for more than 5 consecutive months.


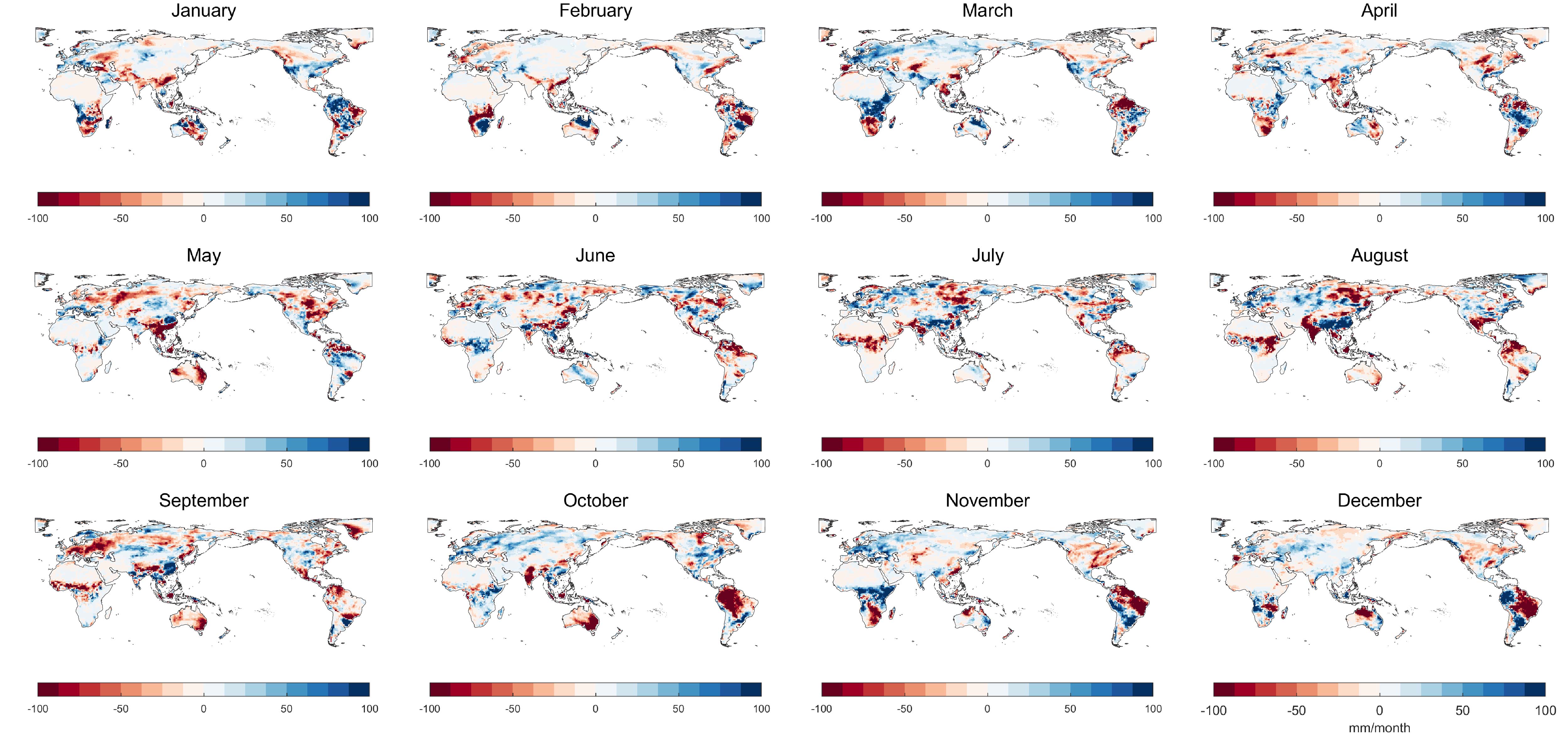


**Supplementary figure 8 Spatial patterns of monthly mean precipitation (Unit: mm/month) in 2023 relative to 2022.**


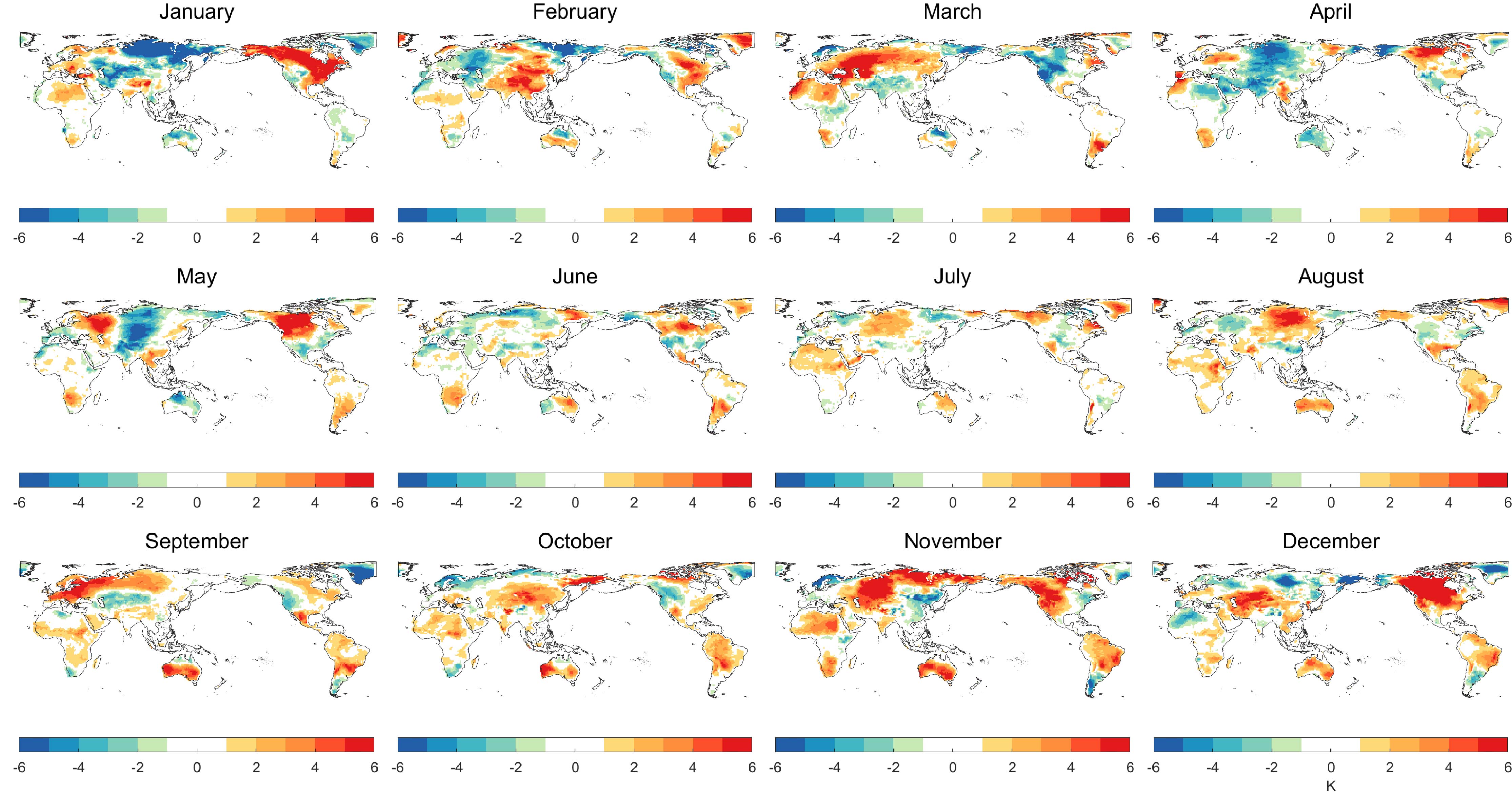


**Supplementary figure 9 Spatial patterns of monthly mean temperature (Unit: K) in 2023 relative to 2022.**


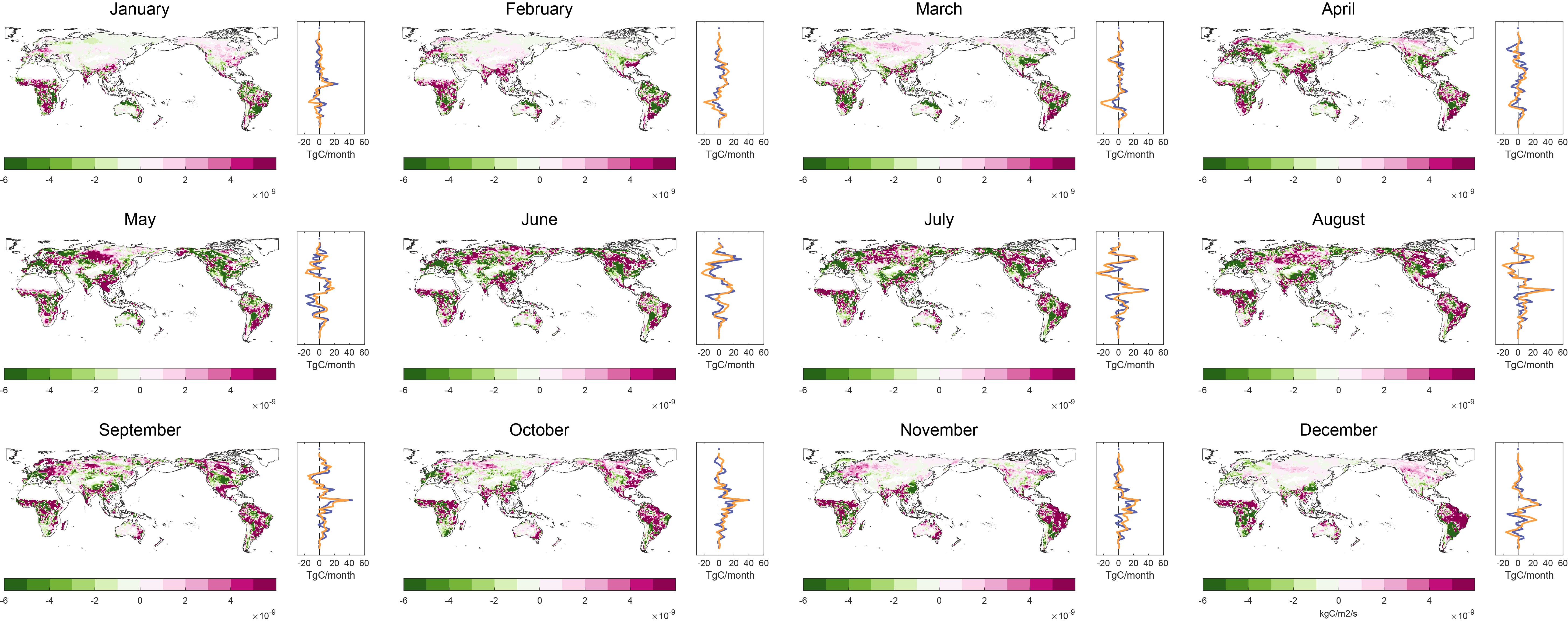


**Supplementary figure 10 Spatial patterns of monthly land carbon fluxes estimated by AI-DGVMs in 2023 relative to 2022.** Main panels show the net land carbon fluxes anomalies estimated by AI-ORCHIDEE and AI-CABLE, and the right panels show the aggregates of every 2° in the latitudinal direction. All positive values in the figure mean more carbon release into the atmosphere.


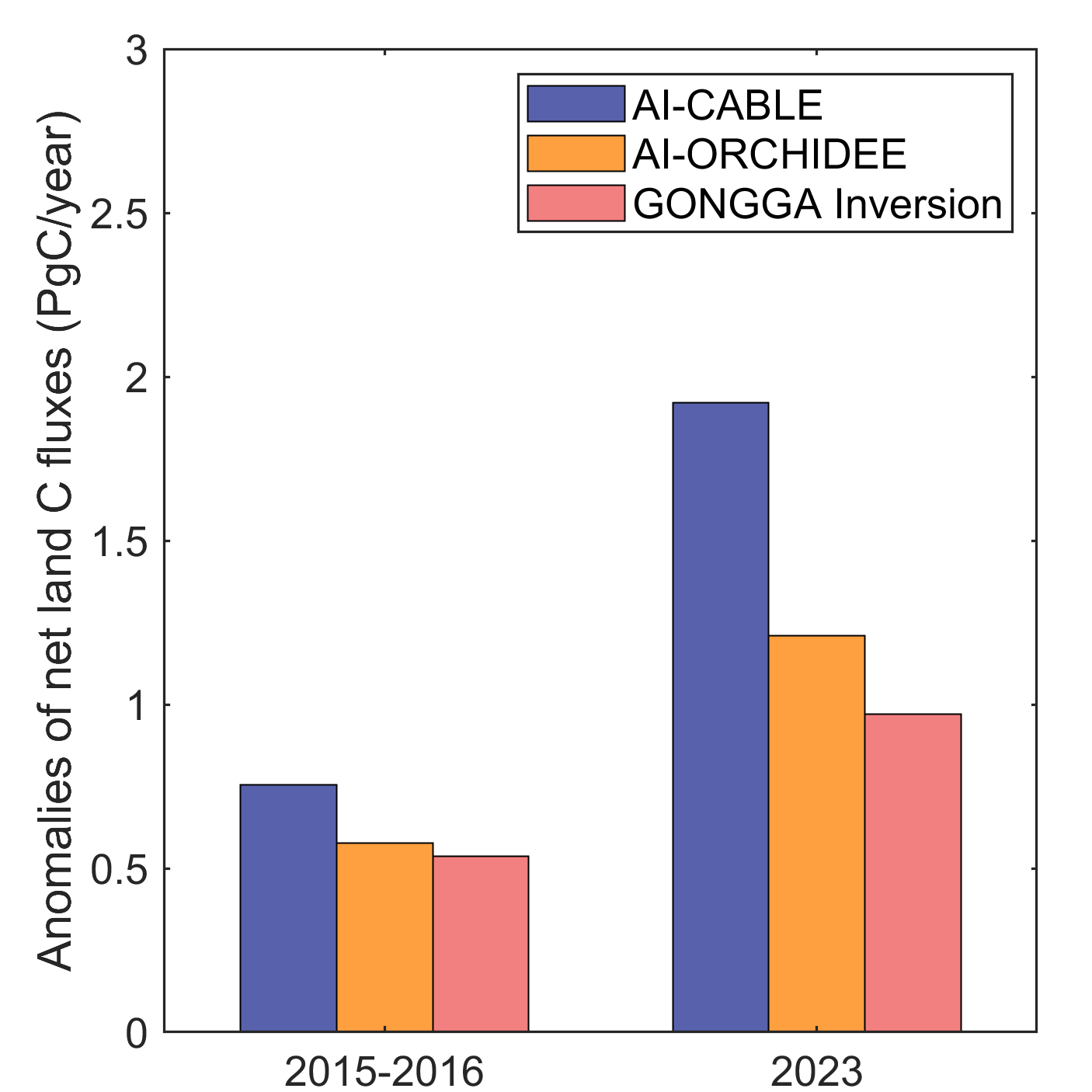


**Supplementary Figure 11 Tropical land carbon sink decline during 2015-2016 and 2023 El Niño events.** We detrend land carbon flux estimated from AI-CABLE, AI-ORCHIDEE, and GONGGA inversion for each grid from 2015 to 2023, then summed all grids in tropics to calculate the annual land carbon flux anomalies. For the 2015-2016 event, we averaged the two-year anomalies. Positive values indicate increased net carbon release or decreased net carbon uptake.


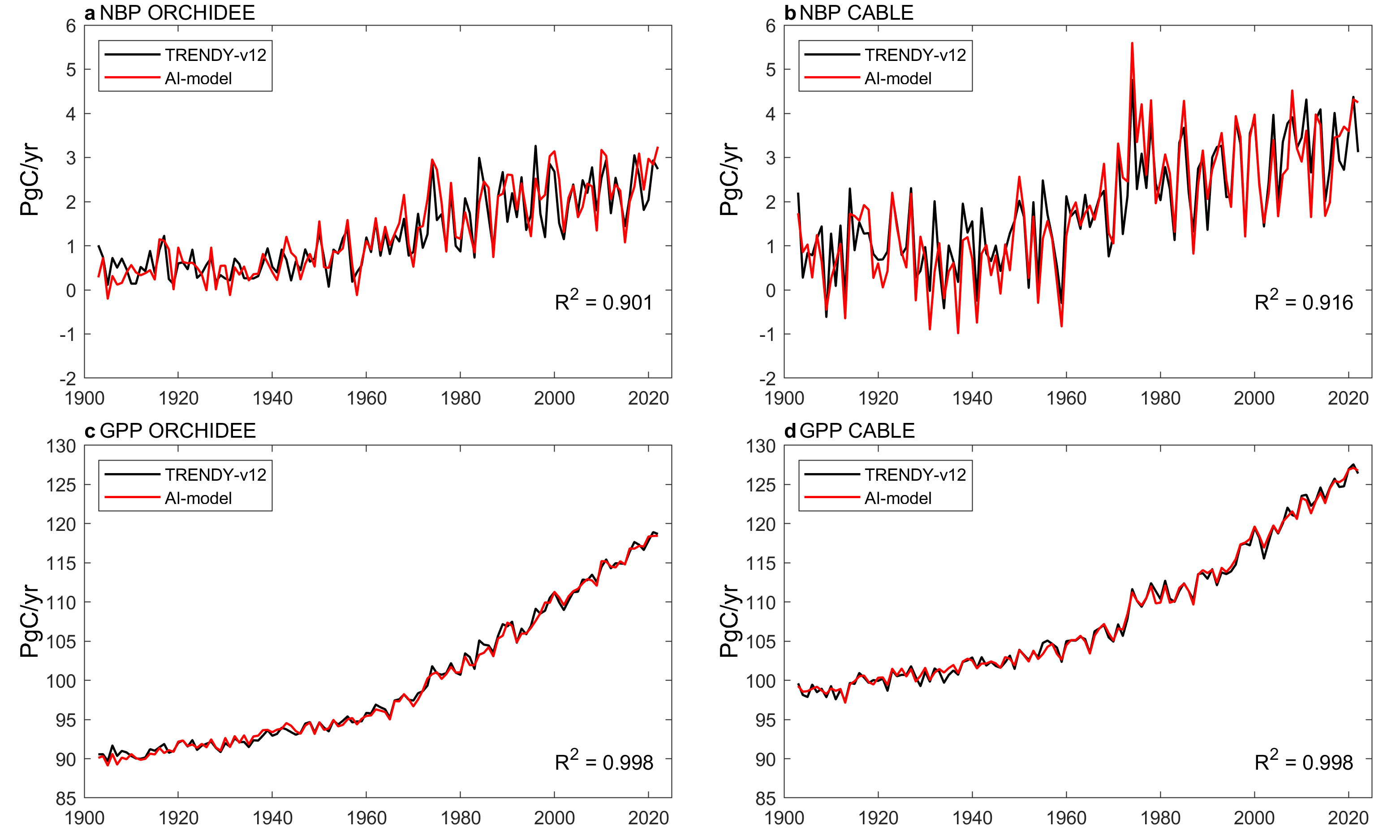


**Supplementary Figure 12 Variations of global NBP and GPP during 1903−2022 estimated by TRENDY-v12 models and AI models.** We pooled the prediction results of 5 ensemble member altogether and composed AI-predicted estimates of 120 years, and compared with TRENDY-v12 source estimates for verification.


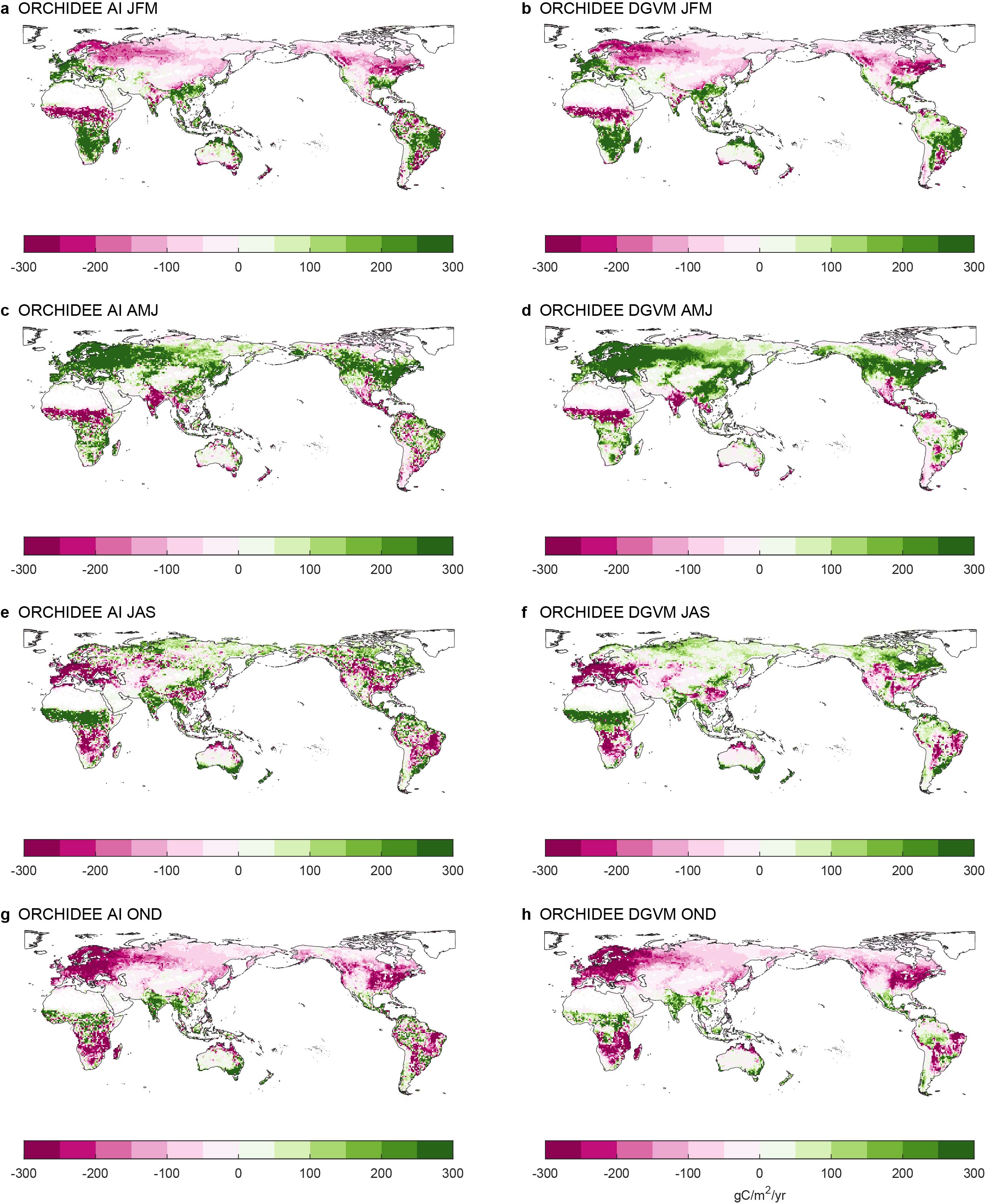


**Supplementary figure 13 Spatial patterns of seasonal mean NBP in 2022 estimated by AI-ORCHIDEE and TRENDY-v12.** Left and right columns show the comparison of ORCHIDEE between the AI model and the TRENDY estimates every 3-month mean, respectively, where JFM refers to the average of January to March, with AMJ, JAS, and OND similar. The setting of the AI estimates is described in Supplementary Table 1.


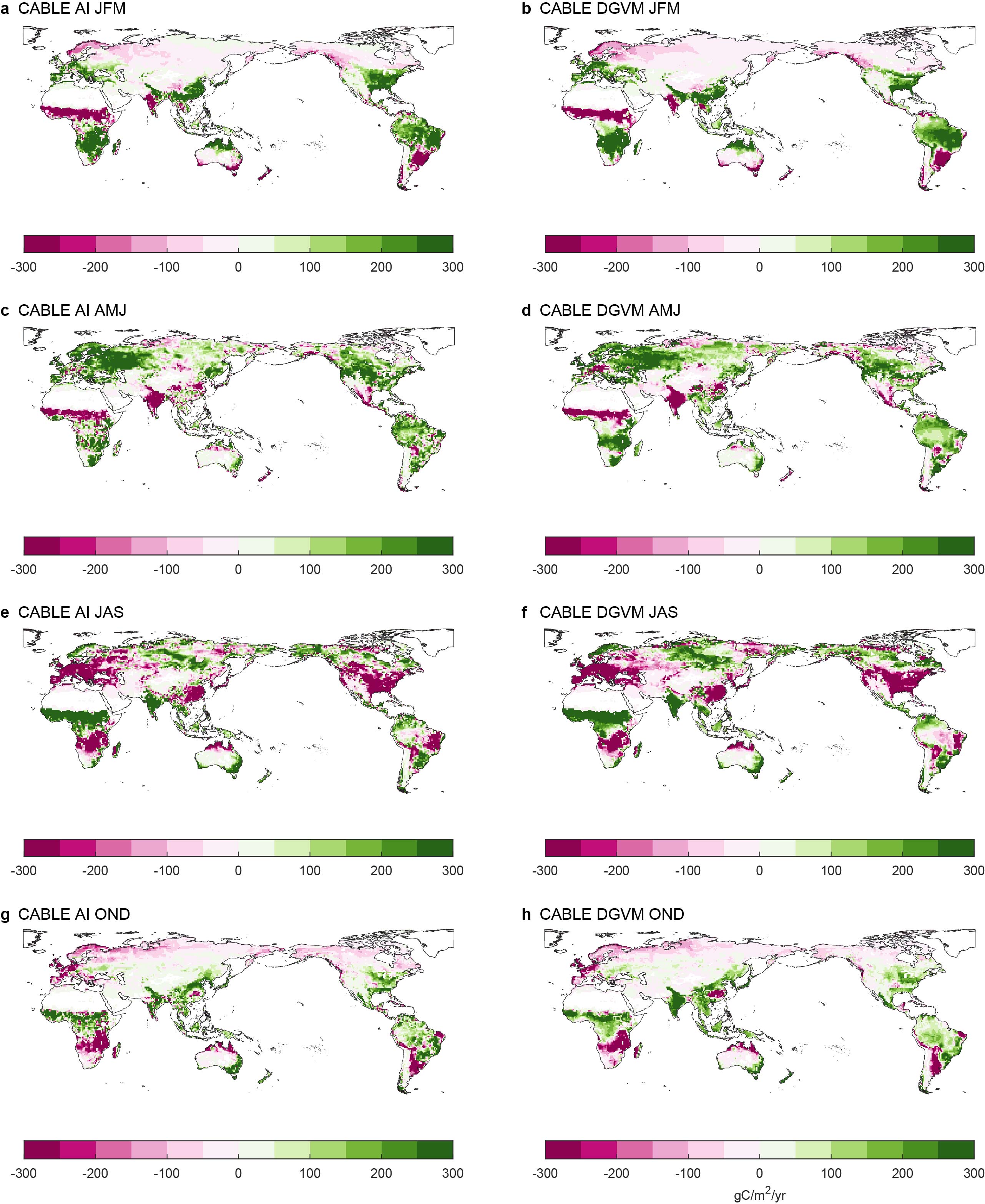


**Supplementary figure 14 Spatial patterns of seasonal mean NBP in 2022 estimated by AI-CABLE and TRENDY-v12.** Left and right columns show the comparison of CABLE between the AI model and the TRENDY estimates every 3-month mean, respectively.


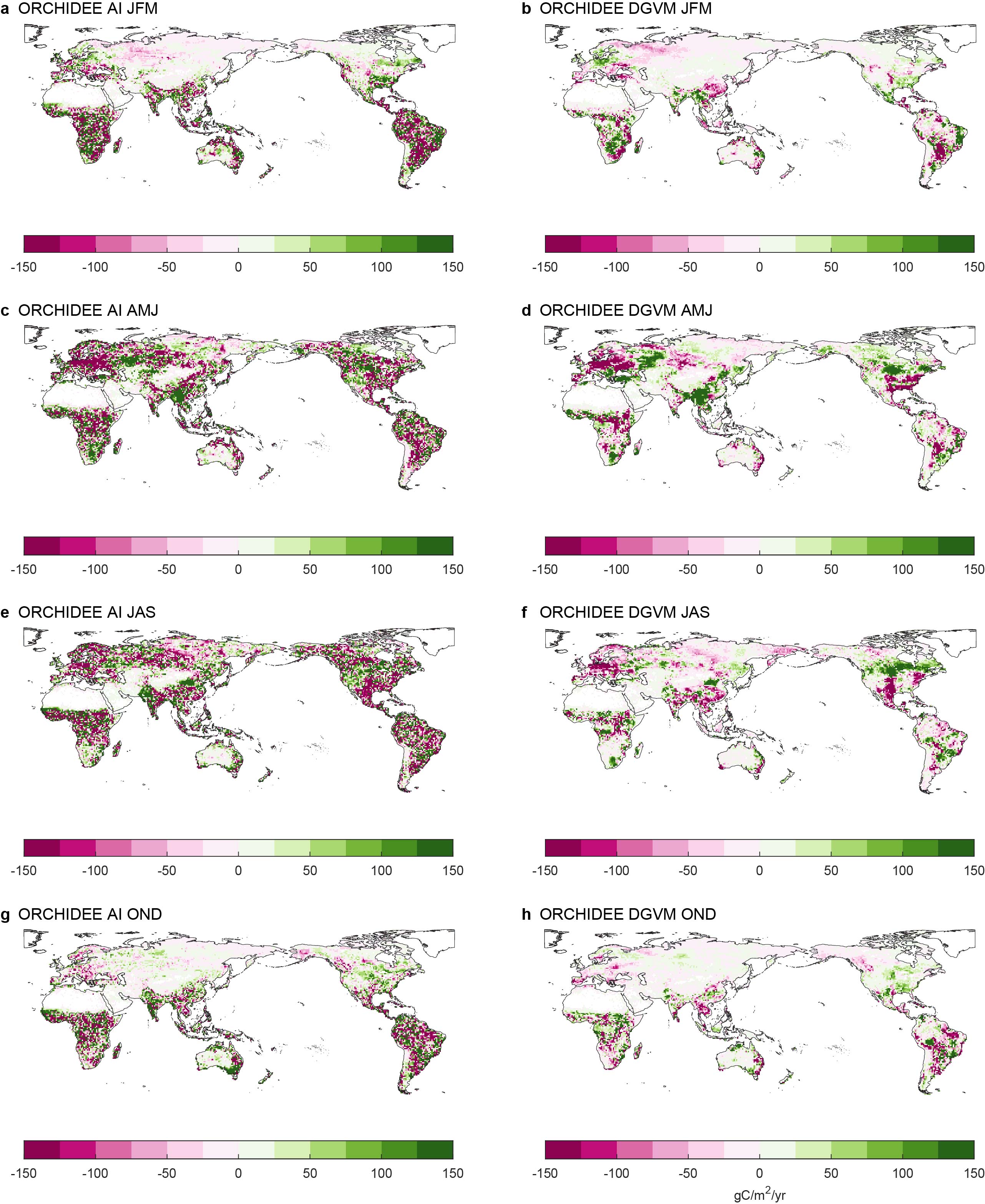


**Supplementary figure 15 Spatial patterns of seasonal mean NBP in 2022 relative to 2021 estimated by AI-ORCHIDEE and TRENDY-v12.** Left and right columns show the comparison of ORCHIDEE between the AI model and the TRENDY estimates every 3-month mean, respectively.


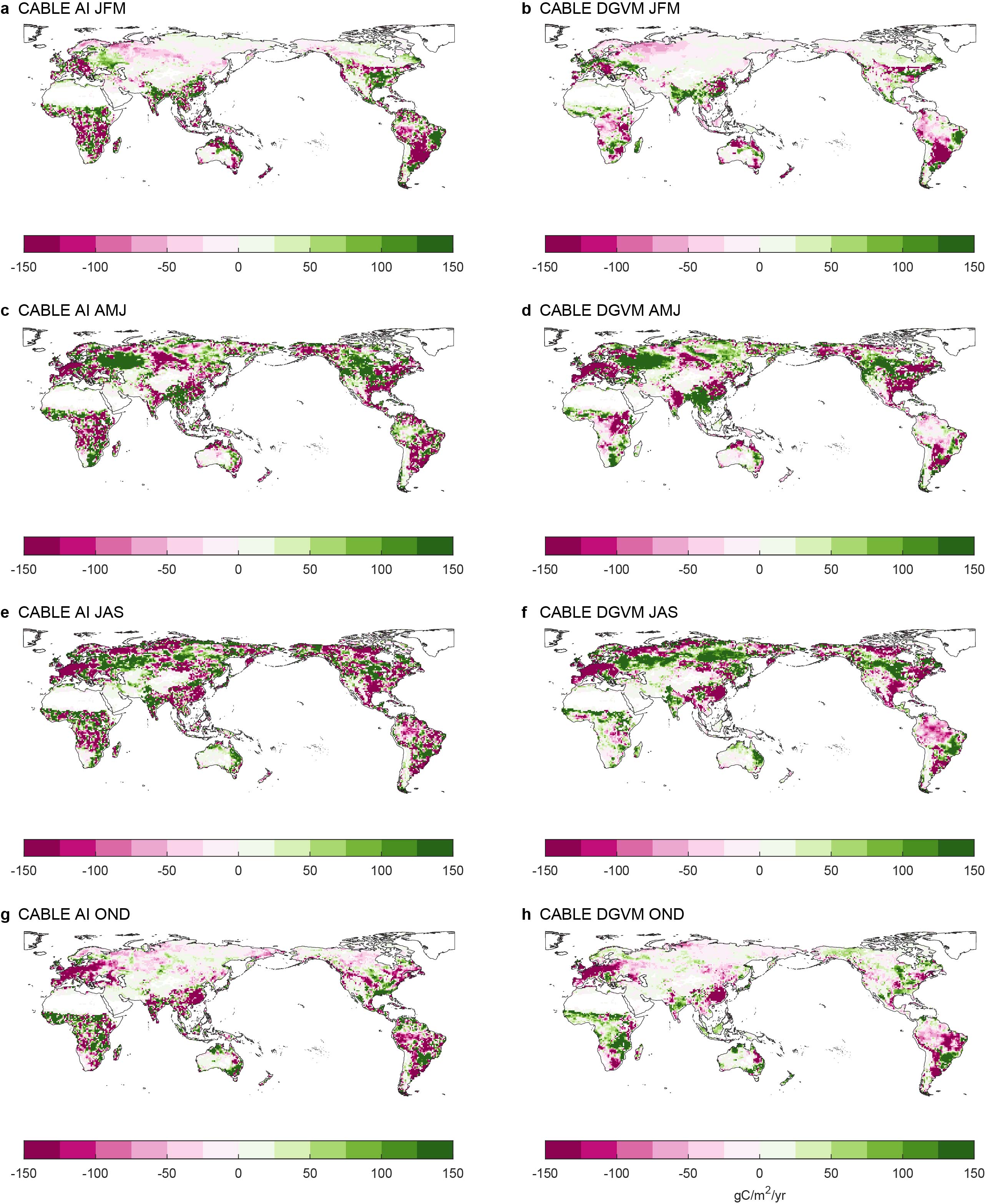


**Supplementary figure 16 Spatial patterns of seasonal mean NBP in 2022 relative to 2021 estimated by AI-CABLE and TRENDY-v12.** Left and right columns show the comparison of CABLE between the AI model and the TRENDY estimates every 3-month mean, respectively.
